# Liver Transplantation and Alcoholism Rehabilitation

**Published:** 1994

**Authors:** Thomas Beresford

**Affiliations:** Thomas Beresford, M.D., is a professor of psychiatry at the University of Colorado School of Medicine, Veterans Affairs Alcohol Research Center, University of Colorado Alcohol Research Center, Denver, Colorado

In the excitement of finding a remarkably high rate of first-year abstinence among his alcoholic liver transplant patients (Starzl et al. 1988), Dr. Thomas Starzl, the pioneer of transplant surgery in this country, commented to the press that liver transplantation might be the ultimate cure for alcoholism.^1^ His study of transplanted alcoholic patients was published with little comment on methods of patient selection or of posttransplant care. Five years later Starzl and colleagues presented data that argued the opposite case—that those with alcoholic hepatitis and cirrhosis show remarkably high rates of relapse to uncontrolled drinking despite having undergone liver transplantation (see Bonet et al. 1993). How can one find a rational approach between these two extremes? The best answer is a complex one, requiring a careful understanding of the methods of preoperative patient selection and of postoperative care. This article offers a brief overview of the topic; for more detail, see Lucey et al. 1994.

Table 1 lists the data from four liver transplant programs. These programs have reported 1-year abstinence rates among liver transplant recipients who also suffered from preexisting alcohol addiction. All programs reported first-year abstinence rates that approximated 90 percent, a remarkably high frequency when compared with the 30- to 50-percent range reported in alcoholism treatment studies that did not involve a procedure as drastic as liver transplantation (Moos 1990; Vaillant 1983). On the surface, it is easy to conclude that a chronic life-threatening illness, followed by the extreme stress of a lengthy operation and its ensuing recovery, might deter a patient from future drinking. There is the added implication that the patient will not receive another transplant if drinking begins again and results in a second liver failure.

A closer look at the programs reveals several common threads. Each program carefully selects and then follows those alcohol-dependent patients for whom the program will agree to provide a liver transplantation. Selection is based in part on the perceived risk that a particular patient will return to uncontrolled alcohol use. The University of Michigan’s liver transplant program has led in the development of selection procedures for alcoholic transplant candidates (Beresford et al. 1990), and each of the other programs incorporates some aspects of these procedures in their own formulations. However, the questions arise: Are there empirical guidelines for predicting long-term remission from alcohol dependence? In particular, does the transplant itself have a positive effect on maintaining abstinence? Currently, there are only partial answers to these questions, which are discussed below.

## Predicting Abstinence

Research has shown the following characteristics among patients who are likely to maintain long-term abstinence: (1) self-recognition of alcohol dependence and acceptance of it as a condition to be dealt with, (2) a socially stable living environment, (3) freedom from severe psychiatric disorders, and (4) available resources that facilitate continued abstinence (Beresford 1990; Lucey et al. 1994).

Vaillant’s work (1983) is especially pertinent. In an 8-year prospective^2^ study, he noted that alcoholics who had been abstinent for 3 years or longer had at least two of four clinical indicators. First, they structured their time with substitute activities that limited the potential time they could spend drinking. Second, they had developed a relationship with a person committed to their well-being who put clear limits on his or her toleration of their drinking. Third, they found a sense of hope or of improved self-esteem in some aspect of their lives that counteracted the often intense guilt they felt as a result of their pathological alcohol use. Fourth, they suffered some noxious consequence of drinking, such as severe abdominal pain from pancreatic inflammation or an ethanol-disulfiram reaction^3^ (see the article by Anton, pp. 265–271).

As most liver transplant programs now realize, alcoholic candidates who recognize their alcohol dependence as a serious and continuing health risk, who have a socially stable environment, and who possess most or all of the factors described by Vaillant are unlikely to relapse to alcoholic drinking during the first 12 months after a liver transplant. However, it is not certain whether these factors are the actual cause of relapse prevention in these patients.

For most liver transplant recipients, all the predictive factors that Vaillant elucidated occur in the natural course of postoperative care during the first year (Beresford et al. 1992). For example, the thought of death as a direct and negatively perceived consequence of drinking may reinforce abstinence, at least in the months immediately following the transplant procedure. For most patients, the care of the new liver requires a nearly ritualized ingestion of anti-immune medicines twice daily; along with postsurgery rehabilitation, this becomes a substitute activity that structures time and serves to replace drinking as a primary concern in life. Continued contact with the transplant team postoperatively offers a rehabilitation relationship that bolsters the patient’s self-esteem and reinforces the necessity of abstaining from alcohol. Finally, liver transplantation offers a profound sense of hope in providing a second chance at life for an alcoholic whose fate otherwise is certain death. This too results in an improved sense of self-worth for most alcoholic recipients. It is therefore not surprising that most properly selected alcohol-dependent liver transplant recipients remain abstinent without exposure to formal alcoholism rehabilitation programs either before or after the operation.

## Long-Term Abstinence

All the patient characteristics described by Vaillant (1983) as favorable to abstinence may be fostered naturally as the transplant team assists the patient in the physical recovery process. However, the frequency and intensity of each patient’s contact with the team can be expected to decrease during the course of recovery from the operation. Discussed below is the course of postoperative adjustment among alcoholics beyond the first year and its implications for sustained abstinence.

The Michigan group (Campbell et al. 1993) provided a brief report on a series of 52 alcohol-dependent patients who had undergone liver transplantation. All subjects were rigorously selected and were considered to be at low risk for alcoholism relapse after surgery. Contrary to the practice at some other programs, however, no fixed period of preoperative sobriety was required. Thirteen patients who died within 6 months from causes not related to alcohol use were excluded from analysis, along with one patient whose preoperative evaluation was not available. The final study group included 38 patients, who were followed for 36 months on average. Statistical analysis determined the likelihood of posttransplant total abstinence to be 92 percent after 1 year and 74 percent after both 2 and 3 years.

During the 3-year term, the majority (69 percent) of subjects remained completely abstinent, whereas an additional minority (18 percent) experienced brief drinking relapses. The latter group represented seven patients who reported limited alcohol consumption for brief periods that did not result in injury, medical complication, or a return to uncontrolled alcohol use. The overall lack of alcoholic relapse and injury in these two groups seemed to be due, in part, to attentive long-term followup care.

A small minority (13 percent) of the subjects returned to uncontrolled drinking over the 3-year period. This group included a total of five liver transplant recipients who had suffered severe alcoholic relapses requiring medical hospitalization; one of them died from transplant rejection because of poor compliance with the anti-immune medicines while drinking. This number of seriously relapsing alcoholics was too small to determine whether relapse could be predicted by pretransplant factors, such as length of sobriety before the initial evaluation. The problem of insufficient numbers of subjects also has hampered other attempts to evaluate the predictive value of such factors (Osorio et al. 1994). Nevertheless, the low rates of severe alcoholism relapse even after 3 years is noteworthy. It argues for continued allocation of liver transplants to carefully selected alcohol-dependent candidates and indicates the need to improve predictive and followup methods.

Both 1- and 3-year relapse rates among alcohol-dependent liver transplant recipients appear to be significantly lower than those reported among nonselected patients attending alcoholism rehabilitation programs. The best 1-year rates of abstinence among the latter reported in the literature are in the range of 50 percent (Vaillant 1983; Moos et al. 1990), less than the 3-year rate noted above among the transplant patients. This difference may be explained by the combination of three elements: patient selection, the transplant experience, and factors that support long-term abstinence. The long-term data from transplant recipients have not been replicated, however, and consist only of a small sample surveyed through followup contacts rather than systematically tracked over time. A larger series of alcohol-dependent liver transplant recipients who have been carefully selected, evaluated, and followed through time is needed to establish high rates of posttransplant abstinence as a firm empirical observation and to begin to determine the reasons for it.

Of the 3,000 liver transplants performed annually in this country, only a minority involve alcohol-dependent patients (Lucey et al. 1994). The number of alcohol-dependent persons in the Nation at any time is estimated to be in the several millions. The following findings can be learned from this small, unique patient group and can be generalized to treating the larger population of problem-drinking Americans:

- Providing drastic or painful medical procedures alone, such as disulfiram treatment, probably will not result in sustained abstinence from alcohol.
- Continuous, active care aimed at providing or nurturing the good outcome factors described by Vaillant probably will result in higher than expected abstinence rates if that care can be integrated into a program of ongoing medical surveillance.
- Patients who demonstrate favorable outcome prediction profiles may require little in the way of formal alcoholism rehabilitation treatment.
- Persons suffering from alcohol dependence whose outcome prediction profiles match those factors associated with sustained abstinence are probably good-risk patients for costly and extraordinary medical procedures of any type.

In the current era of health care reform and reduced spending, some people will argue that alcoholics, by virtue of their alleged vice, do not deserve liver or heart transplantations or other such costly procedures. Actual transplant data argue to the contrary and support the case for regarding alcoholism as resembling any other illness in that it is amenable to diagnosis, predictive assessment, and understanding through the methods of careful clinical science.

## Figures and Tables

**Table 1:** Data on Alcoholic Liver Transplant Recipients From Four Medical Centers

Patient Information	Liver Transplant Centers			

	Baylor^1^	Pacific^2^	Michigan^3^	UCSF^4^
Number	50	29	39	35
Median Age (years)	49	47	44	47
Gender				
Male (%)	75	64	64	70
Female (%)	25	36	36	30
1-Year Survival (%)	89	94	78	100
1-Year Abstinence (%)	87	83	93	92

1 Baylor University Medical Center, Dallas, TX (Tripp et al. 1992).

2 Pacific Medical Center, San Francisco, CA (Gish et al. 1993).

3 University of Michigan Medical Center, Ann Arbor, MI (Lucey et al. 1992).

4 University of California at San Francisco Medical Center, San Francisco, CA (Osorio et al. 1994).

## Footnotes

1 Throughout this article, terms such as “alcohol addiction,” “alcoholism,” and “problem drinking” are used. These terms overlap in alcoholism literature; therefore, the wording in each case is based on the terms used in the reference cited.

2 A prospective study tracks a group of subjects for a period of time following initiation of the study.

3 Disulfiram (Antabuse^®^) is a medication prescribed to discourage drinking by producing unpleasant symptoms in combination with alcohol.
